# Reversible mislocalization of a disease-associated MRE11 splice variant product

**DOI:** 10.1038/s41598-018-28370-5

**Published:** 2018-07-04

**Authors:** Andrea J. Hartlerode, Joshua A. Regal, David O. Ferguson

**Affiliations:** 1 Department of Pathology, The University of Michigan Medical School, Ann Arbor, MI 48109-2200 USA; 2 Molecular and Cellular Pathology Graduate Program, The University of Michigan Medical School, Ann Arbor, MI 48109-2200 USA

## Abstract

Ataxia-telangiectasia (AT) and related disorders feature cancer predisposition, neurodegeneration, and immunodeficiency resulting from failure to respond to DNA damage. Hypomorphic mutations in *MRE11* cause an AT-like disorder (ATLD) with variable clinical presentation. We have sought to understand how diverse *MRE11* mutations may provide unique therapeutic opportunities, and potentially correlate with clinical variability. Here we have undertaken studies of an *MRE11* splice site mutation that was found in two ATLD siblings that died of pulmonary adenocarcinoma at the young ages of 9 and 16. The mutation, termed *MRE11* alternative splice mutation (*MRE11*^ASM^), causes skipping of a highly conserved exon while preserving the protein’s open reading frame. A new mouse model expressing *Mre11*^ASM^ from the endogenous locus demonstrates that the protein is present at very low levels, a feature in common with the *MRE11*^ATLD1^ mutant found in other patients. However, the mechanisms causing low protein levels are distinct. MRE11^ASM^ is mislocalized to the cytoplasm, in contrast to MRE11^ATLD1^, which remains nuclear. Strikingly, MRE11^ASM^ mislocalization is corrected by inhibition of the proteasome, implying that the protein undergoes strict protein quality control in the nucleus. These findings raise the prospect that inhibition of poorly understood nuclear protein quality control mechanisms might have therapeutic benefit in genetic disorders causing cytoplasmic mislocalization.

## Introduction

Inherited deficiency in the ability of cells to respond to DNA damage leads to instability of the genome. DNA double-strand breaks (DSBs) are a highly toxic form of damage, as both strands of the double helix are disrupted. Syndromes resulting from defective DSB responses feature diverse sequelae such as cancer, immunodeficiency, neurodegeneration and developmental delay^1^. To varying degrees these conditions impact the general population at later ages. Therefore, study of inherited DSB repair syndromes helps shed light on common health challenges.

**Figure 1:**
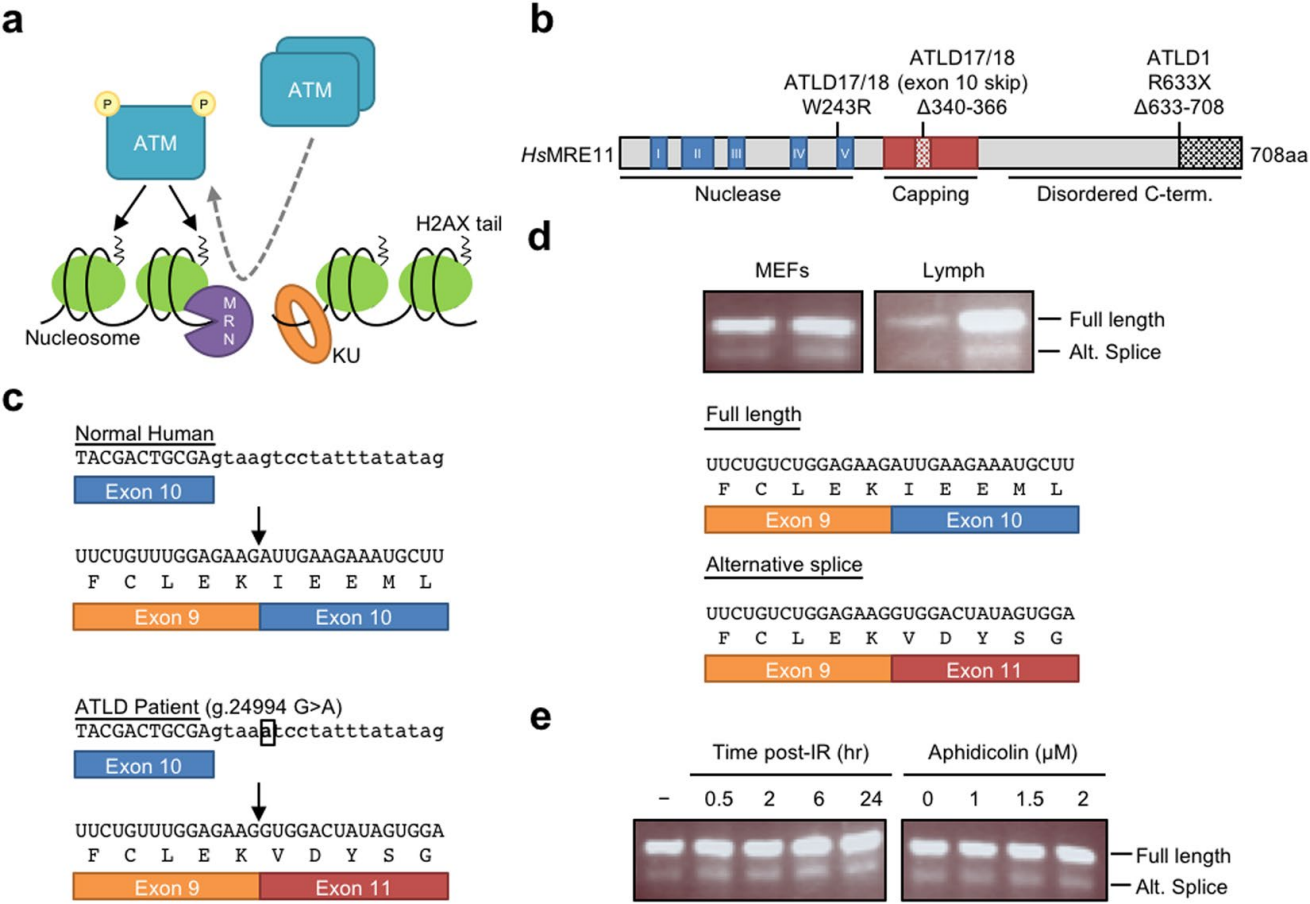
*MRE11*^ATLD17/18^ alternative splice mutant (ASM) resembles a murine endogenous alternative splice variant. (**a**) Schematic of DNA double-strand break sensing by MRN and KU complexes, and subsequent initiation of DNA damage signaling by the ATM kinase. P, phosphate. Activation of the ATM kinase results in dissociation of an inactive ATM dimer into an autophosphorylated active monomer. ATM phosphorylates the tail of histone variant H2AX (and other substrates not shown), driving changes to local chromatin. See text for further details. (**b**) Stick diagram of *Hs*MRE11 with pertinent domains and ATLD disease mutations labeled. (**c**) Sequences of genomic DNA (exon uppercase, intron lowercase), mRNA, and protein from the normal human *MRE11* allele and the mutant human allele in *MRE11*^ATLD17/18^ patients in which a single base substitution (black box) in intron 10 leads to skipping of exon 10 during RNA splicing. (**d**) RT-PCR using a forward primer in exon 9 and a reverse primer in exon 11. A smaller product resulting from exon 10 skipping is detected in both mouse embryonic fibroblast (MEF) and lymphocyte RNA. The mRNA and predicted protein sequence for full-length and alternatively spliced murine *Mre11* are shown below. (**e**) RT-PCR described in (**d**) reveals that exposure to ionizing radiation (IR) or aphidicolin does not alter the relative amount of alternatively spliced *Mre11* in MEFs. RT-PCR agarose gel images are cropped images. The full-length images can be found in Supplementary Fig. S3.

The cellular response to DSBs in mammals features an initial recognition of damage, followed by signaling cascades that trigger cell cycle checkpoints and other changes to cellular behavior. The DNA ends are recognized and bound by two multiprotein complexes - MRE11/RAD50/NBS1 (MRN) and KU70/80 (KU) (Fig. 1a). A poorly understood interplay between these complexes facilitates recruitment and activation of the ataxia-telangiectasia mutated (ATM) kinase, considered the master controller of DSB responses^2,3^. ATM phosphorylates proteins in local chromatin to affect large-scale changes that provide a scaffold for assembly of higher-order complexes that protect, process, and ultimately repair the DNA^4^. These events occur with remarkable speed, and can be detected within 15 seconds of experimentally-induced damage^3^.

Mutation of *ATM* causes ataxia-telangiectasia (AT) (MIM 208900), a syndrome featuring neurodegeneration, cancer predisposition and immune dysfunction^5^. Given the functional relationship between ATM and MRN, it follows that mutations in this complex cause similar syndromes. For example, biallelic mutation in *NBS1* results in Nijmegen breakage syndrome (NBS) (MIM 251260), a disease that superficially resembles AT^6^. However, differences are also apparent, the most notable of which is the effect on the brain. Whereas AT patients are usually asymptomatic at birth and develop degeneration of the cerebellum later, NBS patients are commonly born with microcephaly and manifest severe developmental delays. Although most NBS patients carry a single founder mutation (657del5), an NBS-like patient was found to have biallelic mutation in *RAD50*^7,8^.

The MRE11 component of MRN forms a heterotetramer with RAD50 that binds DNA at DSBs and tethers DNA ends in close proximity^9^. Within this structure, MRE11 catalyzes endo- and exonuclease activities that process DNA ends in the early stages of repair. The nuclease activities are provided by an N-terminal domain that is highly conserved in MRE11 from all species. In eukaryotes, there are several hundred additional C-terminal amino acids that are poorly conserved and without known catalytic activity (Fig. 1b). ATLD results from a diverse group of MRE11 mutations that are spread throughout the protein^10^. This distinction from NBS was apparent beginning with the first report of inherited *MRE11* mutations^11^. Several patients diagnosed clinically with AT, displaying cerebellar degeneration, cellular hypersensitivity to ionizing radiation (IR), and chromosome instability, were found to have no alteration in *ATM* DNA sequence or protein expression. This lead to the disease name ataxia-telangiectasia-like disorder (ATLD) (MIM 604391). Further study revealed two independent partial loss-of-function *MRE11* mutations. One was a nonsense mutation (R633X) that deletes 78 amino acids from the C-terminus (Fig. 1b), while the other was a missense mutation in the N-terminal nuclease domain (N117S)^11^.

While syndromes resulting from mutations in MRN components bear many similarities, differences in clinical outcomes are also apparent. ATLD displays post-natal cerebellar degeneration, in contrast to the microcephaly of NBS^1^. There may also be an important difference in the degree of cancer predisposition. Whereas NBS patients have strong predisposition to multiple types of cancer, the first described ATLD patients have not yet been reported to develop cancer^11^. This varied clinical presentation may be due to the requirement for mutations in MRN components to be hypomorphic, as nullizygosity causes early embryonic lethality in mice^12–14^. The location of the mutations and the impact of each disease allele may therefore be diverse, requiring only enough function to support viability. In this regard, we have previously shown that MRE11 nuclease activity is essential for early embryogenesis in mice^12^. Therefore, at least some nuclease activity must be preserved for viability.

Highlighting the variable clinical course is a more recent report of two brothers with ATLD who did succumb to cancer. These cases (termed ATLD17/18) are striking in that both brothers died of pulmonary adenocarcinoma at the young ages of 9 and 16^15^. While independent cancer modifiers cannot be ruled out, the fact that the ATLD17/18 patients were compound heterozygotes for novel *MRE11* mutations (one from each parent) supports the notion that cancer resulted from unique aspects of these disease alleles. This idea is reinforced by the lack of cancer in the siblings and parents, whom each harbored one *MRE11* mutation paired with a wildtype allele. One of the mutations (c.727 T > C; W243R) occurs at a highly conserved residue near motif V of the N-terminal nuclease domain which is important for maintaining both the structure of MRE11 and its interaction with NBS1^10,16^ (Fig. 1b). The second mutation is more complex, and is a single nucleotide substitution in an intron near a splice donor site (g.24994 G > A). The authors reported that this mutation causes skipping of the 81-nucleotide exon 10. The resulting 9-to-11 splice variant maintains the open reading frame, and is predicted to encode MRE11 lacking 27 amino acids situated in a structural feature unique to MRE11 (Fig. 1b,c). This “capping domain” has been shown to make contacts with the 3′-ssDNA tail of a branched DNA substrate, and has been hypothesized to modify the substrate specificity of MRE11^17,18^.

We have recently begun studies to understand if and how varied *MRE11* mutations relate to the diverse clinical outcomes of ATLD^10^. In this study we sought to gain further understanding along these lines and investigate if varied mutations present different therapeutic opportunities. To this end we conducted studies of the *MRE11* exon 9–11 splice variant found in the ATLD17/18 patients. We have generated mice and cell lines that express only this variant. Our studies indicate that the splice variant allele encodes an MRE11 protein subject to poorly understood nuclear protein quality control that leads to low protein levels and cellular mislocalization. This quality control mechanism involves the proteasome, for which inhibition restores cellular localization.

## Results

### The *MRE11*^ATLD17/18^ splice variant is detected in normal mouse tissues

In 1996 a GenBank deposition (NM_001310728) documented an Mre11 splice variant derived from a mouse testes cDNA library. This variant corresponds precisely to the splice product associated with the intronic single base substitution (g.24994 G > A) in the ATLD17/18 patients (Fig. 1b,c)^15^. For clarity, we will refer to this form as Mre11 alternatively spliced mutation (*MRE11*^ASM^), since the *MRE11*^ATLD17/18^ patients also harbored the *MRE11*^W243R^ allele. To determine if *MRE11*^ASM^ is present in normal mouse tissues we isolated *Mre11* cDNAs from lymphocytes and embryonic fibroblasts of mice. Indeed, a subset of clones were approximately 80 bases shorter than anticipated. Sequencing revealed that the shorter variants each lacked the 81 bp exon 10, and contained an accurate in-frame splice from exon 9 to 11 (Table 1). To detect the endogenous *Mre11* splice form more precisely we designed an RT-PCR primer pair in exons 9 and 11 that would produce easily distinguishable PCR products with and without exon 10. We detected the isoform lacking exon 10 in mRNA isolated from both lymphocytes and mouse embryonic fibroblasts (MEFs) (Fig. 1d). In each case the shorter form was present at levels far lower than full-length *Mre11* transcript.

**Table 1:** Analysis of cloned *Mre11* cDNAs.

Sequence	MEFs	B lymphocytes
Exons 9-10-11	6	5
Exons 9–11	5	2

Because this *Mre11*^ASM^ splice variant maintains the MRE11 open reading frame, it is possible that the smaller protein product could have a specialized function. Given the many roles of MRE11 in DNA damage responses, we explored the possibility that alternate splicing could be impacted by DNA damage. MEFs were exposed to IR (10 Gy) to induce DNA double-strand breaks, and allowed to recover for 30 minutes to 24 hours. mRNA levels were analyzed by RT-PCR as described above. No obvious change in the ratio of full-length versus 9–11 splice product was observed (Fig. 1e). MRE11 is also involved in responses to replication stress, such as fork stalling or collapse^19,20^. We therefore exposed MEFs to the DNA polymerase inhibitor aphidicolin, but also observed no apparent changes to the splice variant ratio (Fig. 1e).

We next sought to determine if the *Mre11*^ASM^ exon 9 to 11 splice product can be detected in human cells. PCR approaches analogous to those used in mice were performed on cDNA isolated from wildtype fibroblasts and HeLa cells. In no case did we detect the shorter variant using primers that can detect both splice forms, nor was any product seen in the reaction specific to the 9–11 variant (Supplementary Fig. S2 and data not shown). This would suggest that the alternative 9–11 splice is not universal in normal mammalian cells and increases the confidence that the splice variant detected in the ATLD17/18 patients arose due to the intronic mutation. The presence of the variant specifically in some normal human tissues or cell types cannot be ruled out.

### Generation of murine cells expressing approximately normal levels of MRE11^ASM^

We endeavored to gain further understanding of the consequences of exon 10 skipping. Cells from the deceased Japanese ATLD17/18 patients have not been made available for study. However, even if available these would not be informative since they harbored two distinct *MRE11* mutant alleles. We therefore engineered cells that stably express *Mre11*^ASM^ from cDNA at levels approximately equal to endogenous wildtype MRE11 protein. These immortalized MEFs originally contained one endogenous *Mre11* null allele (*Mre11*^−^) and one *Mre11 Cre/LoxP* conditional allele (*Mre11*^cond^) (Supplementary Fig. S1a,b)^12^. Conversion of *Mre11*^cond^ to *Mre11*^−^ generates cells with an endogenous *Mre11*^−/−^ genotype expressing approximately normal levels of MRE11^ASM^ (Supplementary Fig. S1c). Thus, phenotypes uncovered in these cells reflect specific deficiencies of the MRE11^ASM^ protein.

### MRE11^ASM^ is mislocalized to the cytoplasm, which can be corrected by proteasome inhibition

**Figure 2:**
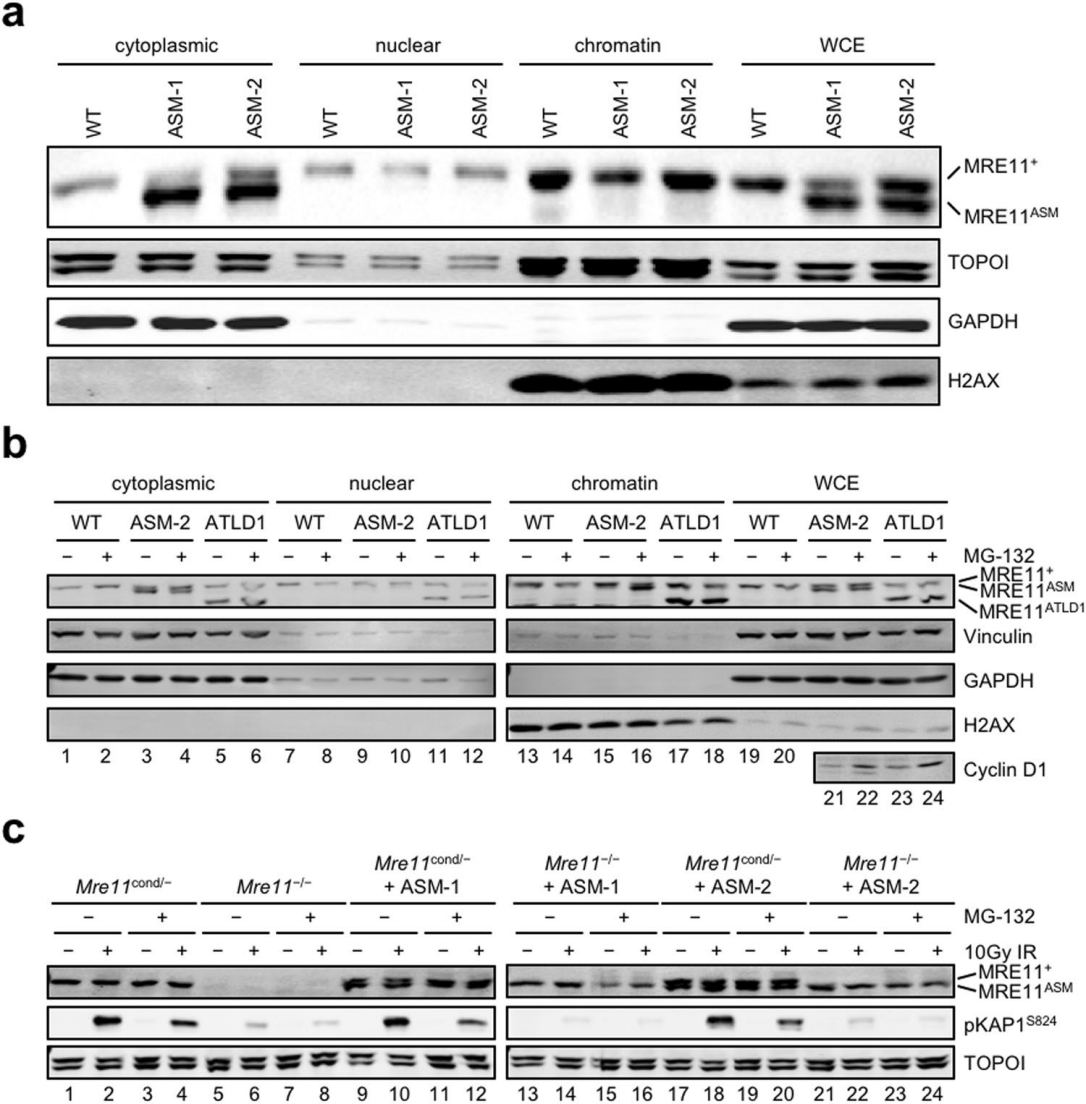
Cellular mislocalization of MRE11^ASM^ and restoration by proteasome inhibition. (**a**–**c**) Immunoblots performed on MEFs, with genotypes and experimental conditions at top, using the indicated antibodies at right. (**a**) Cellular fractionation of wildtype (WT) MEFs and two different MEF clones stably expressing *Mre11*^ASM^ cDNA (ASM-1, ASM-2) reveals that MRE11^ASM^ protein is mislocalized to the cytoplasmic fraction, while wildtype MRE11 (MRE11^+^) is mainly localized in the chromatin fraction. TOPOI, GAPDH, and H2AX are shown as fractionation and loading controls. WCE; whole cell extract. (**b**) Treatment of cells with the proteasome inhibitor MG-132 restores chromatin localization of MRE11^ASM^ (lane 16), but does not affect localization of wildtype MRE11 or the C-terminal deletion mutant MRE11^ATLD1^. Vinculin, GAPDH, and H2AX are fractionation and loading controls. Increase in Cyclin D1 levels after MG-132 in WCE demonstrates effective proteasome inhibition. (**c**) Pretreatment of cells expressing only *Mre11*^ASM^ with MG-132 does not rescue ATM activation as measured by phosphorylation of KAP1 (compare lane 14 to 16 and 22 to 24). TOPOI is a protein loading control. Immunoblot images are cropped images. The full-length images can be found in Supplementary Fig. S4.

Using our *Mre11*^ASM^ cDNA-expressing MEFs we performed cellular fractionation and compared the localization of endogenous wildtype MRE11 (MRE11^+^) to MRE11^ASM^. MRE11^+^ is predominantly associated with chromatin, with smaller amounts found in the nucleus and cytoplasm^21^. Strikingly, in two independent MEF lines, MRE11^ASM^ was not present in chromatin, and was instead only detected in the cytoplasmic fraction (Fig. 2a). We directly compared this mislocalization to that of MRE11^ATLD1^, which truncates the C-terminal 78 amino acids. Like ATLD17/18 patients, ATLD1 patients express very low levels of MRE11. Therefore we stably expressed *Mre11*^ATLD1^ from cDNA in MEF lines that can be conditionally deleted for endogenous *Mre11*. In this case, MRE11^ATLD1^ localization appeared similar to MRE11^+^, with most protein found in the chromatin fraction, with lower amounts present in cytoplasm (Fig. 2b, lane 17). Therefore, unlike Mre11^ATLD1^, absence from the chromatin fraction is a defining feature of MRE11^ASM^ protein.

We further probed the role of MRE11 cellular mislocalization by inhibiting the proteasome. Parallel cultures were incubated with or without the proteasome inhibitor MG-132 for 8 hours prior to fractionation. In whole cell extracts, there was no apparent impact on overall protein levels of MRE11^+^ or the ATLD variants. However, upon fractionation it became apparent that MG-132 exposure corrected the mislocalization of MRE11^ASM^, causing most of the protein to be chromatin-associated (Fig. 2b, lane 16). No impact on the localization of MRE11^ATLD1^ or MRE11^+^ was observed.

The correction of MRE11^ASM^ localization led us to test if MRE11 function is also restored. We exposed control cells (*Mre11*^cond/−^) and two independent MRE11^ASM^-expressing lines to 10 Gy of IR and compared levels of phosphorylated KAP1 after a 30 minute recovery. KAP1 is phosphorylated by ATM after IR, making it a reliable measure of MRE11-dependent ATM activity^22^. Cells expressing wildtype MRE11 (*Mre11*^cond/−^) showed radiation-induced KAP1 phosphorylation, whereas MRE11 deficiency (*Mre11*^−/−^) did not (Fig. 2c, compare lanes 2 and 6). Cells expressing only MRE11^ASM^ displayed a significant defect in KAP1 phosphorylation (Fig. 2c, lanes 14 and 22). Proteasome inhibition did not restore KAP1 phosphorylation, indicating that phenotypic consequences of MRE11^ASM^ are not solely due to protein mislocalization (Fig. 2c, lanes 16 and 24).

### Generation of a germline mouse *Mre11*^ASM^ allele at the endogenous locus

To gain greater understanding of the biological consequences of MRE11^ASM^ expression, we generated a gene targeted mouse model. The point mutation in the ATLD17/18 patients was intronic and impacted splicing. It is uncertain whether the same impact would be observed in mice. For example, Seckel syndrome can be caused by a similar intronic mutation that causes exon skipping in the *ATR* kinase gene. However, a targeted mouse allele analogous to the human mutation did not impact splicing^23^. Based on this, we opted to take a different approach; eliminate exon 10 entirely within the endogenous *Mre11* locus, to facilitate efficient splicing from exon 9 to 11. This design gives the best potential to mimic the disease allele given evidence that such mutations in humans cause skipping in nearly 100% of mRNA^24^.

**Figure 3:**
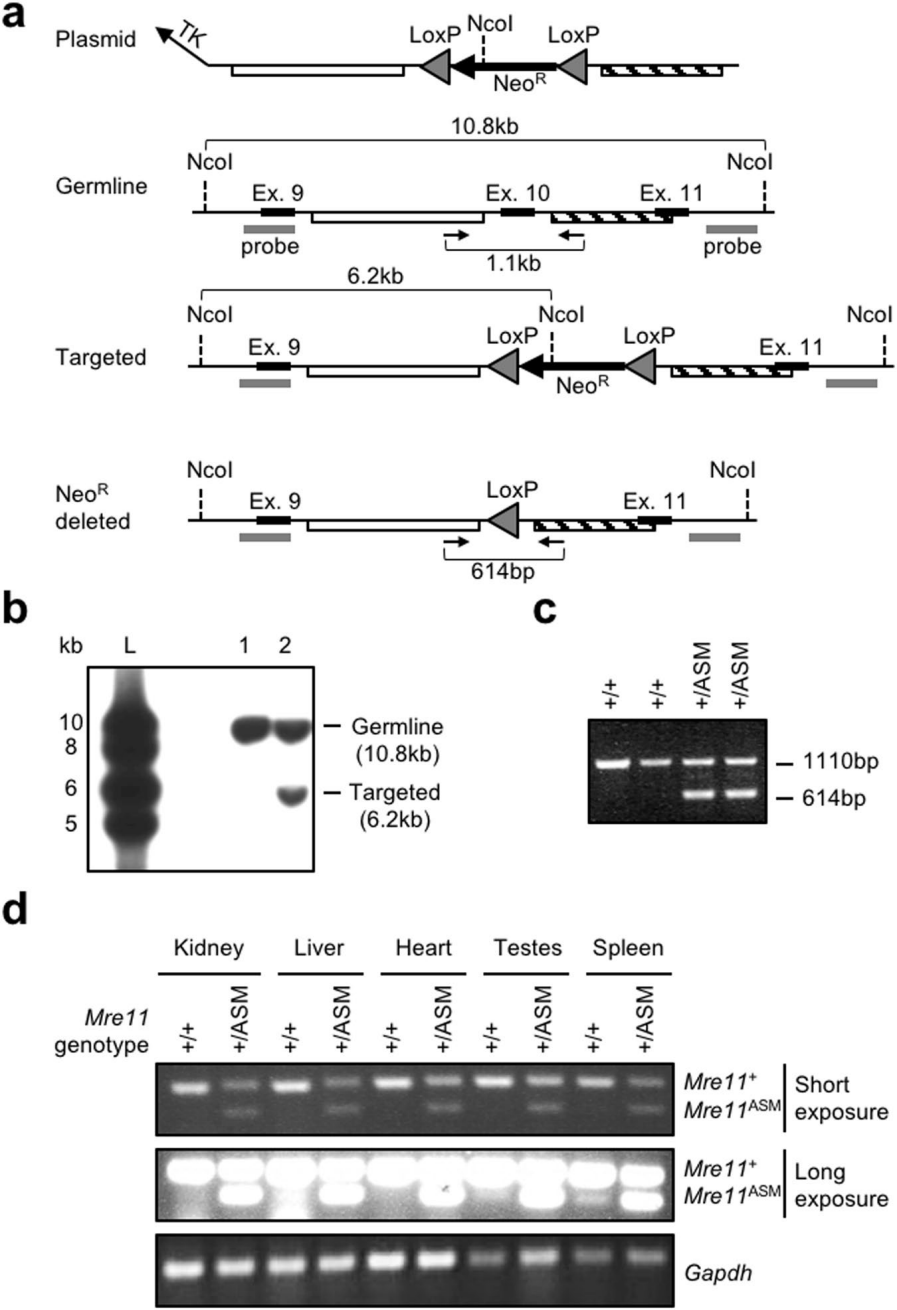
Generation of a germline mouse *Mre11*^ASM^ allele at the endogenous locus. (**a**) *Mre11*^ASM^ targeting strategy. Shown are the targeting plasmid lacking exon 10, germline locus, initial targeted configuration, and targeted locus with *Neo*^*R*^ deleted. Triangles, *LoxP* sites; grey bars, Southern blot probes; black bars, exons; white and striped bars, targeting arms; arrows, PCR genotyping primers. (**b**) Southern blot analysis of a targeted embryonic stem cell clone. NcoI-digested genomic DNA was probed with the 5′ probe depicted in (**a**). Lanes are as follows: 1, parental ES cell line; 2, initial targeted clone; L, ladder. (**c**) Genotyping of germline *Mre11*^+/ASM^ mice. PCR genotyping of genomic DNA from mouse tails was performed with primers depicted in (**a**). *Mre11*^ASM^ produces a smaller band due to loss of exon 10. (**d**) RT-PCR analysis of RNA from germline *Mre11*^+/+^ and *Mre11*^+/ASM^ mice. PCR of cDNA from mouse organs with primers in exon 9 and 11. *Mre11*^ASM^ produces a smaller band due to loss of exon 10 that is readily detectable in heterozygous (*Mre11*^+/ASM^) tissues (see short exposure), and also as a minor product in several wildtype (*Mre11*^+/+^) tissues (see long exposure). Southern blot, genotyping, and RT-PCR agarose gel images are cropped images. The full-length images can be found in Supplementary Fig. S5.

We generated a homologous recombination targeting construct that replaces murine *Mre11* exon 10 along with 181 intronic bases upstream and 234 downstream (Fig. 3a). Initial targeting removed this sequence and introduced a *LoxP*-flanked neomycin cassette that was subsequently removed in the germline by breeding to mice containing the EIIa-Cre transgene (Fig. 3b)^25^. This resulted in a 5.86 kb intron between exons 9 and 11, which was confirmed by PCR (Fig. 3c) and sequencing (not shown).

Early heterozygous mice (*Mre11*^+/ASM^) were sacrificed and dissected to isolate RNA from various tissues. We determined whether our engineered allele successfully permitted splicing from exons 9 to 11 by using the RT-PCR approach described above that detected low levels of endogenous 9–11 splice in mouse fibroblasts and lymphocytes. From all tissues tested we readily detected the shorter form of *Mre11* RNA at levels significantly higher than observed in mice without the *Mre11*^ASM^ allele (Fig. 3d). This PCR product was confirmed by sequencing to be an accurate 9–11 slice variant (not shown).

*Mre11*^+/ASM^ mice were interbred with the goal of obtaining *Mre11*^ASM/ASM^ offspring. After greater than 150 births, no *Mre11*^ASM/ASM^ pups were identified while *Mre11*^+/ASM^ and *Mre11*^+/+^ offspring were obtained with expected Mendelian ratios (Table 2). Therefore homozygosity for the *Mre11*^ASM^ allele causes lethality during embryogenesis. Isolation of embryos at day e13.5 or e11.5 of the mouse gestational period yielded only *Mre11*^+/+^ and *Mre11*^+/ASM^ embryos, indicating that *Mre11*^ASM^ homozygosity causes death early in development (Table 2).

**Table 2:** *Mre11*^ASM/ASM^ causes early embryonic lethality.

Offspring genotypes	Expected	Actual	Expected if embryonic lethal
**Live births**			
*Mre11* ^+/+^	45	60	59
*Mre11* ^+/ASM^	90	118	118
*Mre11* ^ASM/ASM^	45	0	0
Total	180	178*	177
**e13.5 embryos**			
*Mre11* ^+/+^	11	15	15
*Mre11* ^+/ASM^	22	29	30
*Mre11* ^ASM/ASM^	11	0	0
Total	44	44*	45
**e11.5 embryos**			
*Mre11* ^+/+^	15	17	20
*Mre11* ^+/ASM^	30	42	40
*Mre11* ^ASM/ASM^	15	0	0
Total	60	59*	60

Cross: *Mre11*^+/ASM^ × *Mre11*^+/ASM^.

*Χ^2^ p < 0.005.

### Expression from the murine *Mre11*^ASM^ allele

**Figure 4:**
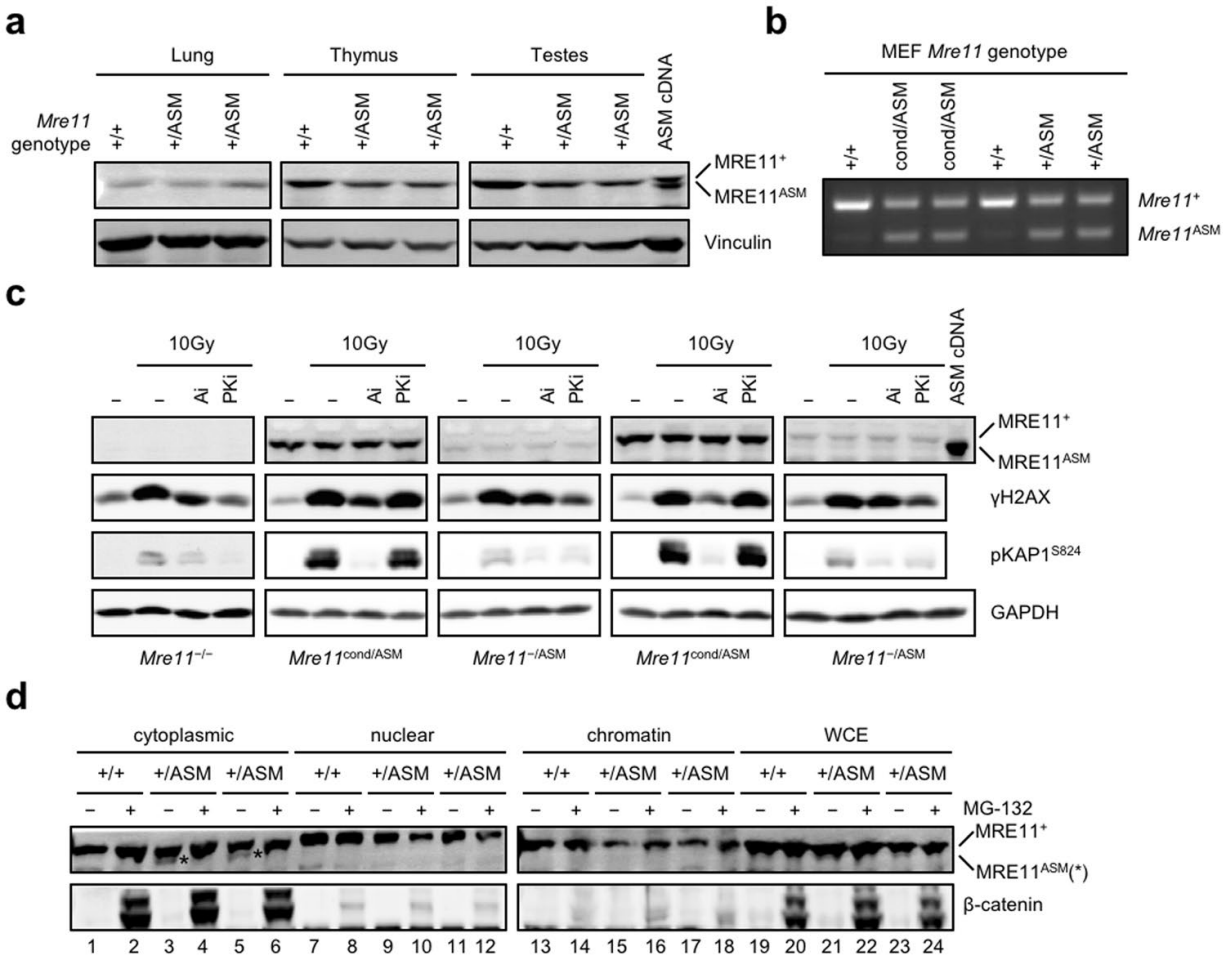
MRE11^ASM^ encoded by the endogenous locus is subject to stringent protein quality control. (**a**) Western blot analysis of proteins isolated from the lung, thymus, and testes of *Mre11*^+/+^ or *Mre11*^+/ASM^ mice. Right lane shows migration of MRE11^ASM^ protein from cells transfected with *Mre11*^ASM^ cDNA. Vinculin is protein loading control. (**b**) RT-PCR analysis of RNA from MEFs derived from germline *Mre11*^*+/+*^, *Mre11*^+/ASM^, and *Mre11*^cond/ASM^ mice. (**c**) Immunoblot comparing levels of DNA damage kinase substrates γH2AX and pKAP1^S824^ 30 minutes post-IR after pretreatment ± kinase inhibitors (Ai - ATM, PKi - DNA-PK) in MEFs harboring a germline *Mre11*^ASM^ allele. GAPDH is protein loading control. MEF genotypes shown at bottom. (**d**) Cellular fractionation of wildtype (*Mre11*^+/+^) and two independently isolated heterozygous (*Mre11*^+/ASM^) MEF lines. A small amount of MRE11^ASM^ protein (indicated by * in lanes 3 and 5) is detected in the cytoplasmic fraction in the absence of MG-132, while MRE11^+^ is in all fractions. β-catenin increase demonstrates proteasome inhibition by MG-132. Immunoblot and RT-PCR agarose gel images are cropped images. The full-length images can be found in Supplementary Fig. S6.

Given the severity conferred by *Mre11*^ASM^ homozygosity we analyzed levels of MRE11 protein in *Mre11*^+/ASM^ mouse tissues. In lung, thymus and testes, full-length MRE11 protein was readily detectable by immunoblotting, but we did not observe a protein product corresponding to the anticipated size of MRE11^ASM^ (Fig. 4a). The loss of 27 amino acids is detectable in these conditions, as observed in extracts from cells transfected with an *Mre11*^ASM^ cDNA expression plasmid (Fig. 4a).

We also derived primary heterozygous MEFs harboring the germline *Mre11*^ASM^ allele and either the wildtype (*Mre11*^+^) or functionally equivalent *Mre11 Cre/LoxP* conditional allele (*Mre11*^cond^)^12^. *Mre11*^ASM^ mRNA was readily detected in both cell types (Fig. 4b). However, like mouse tissues, MRE11^ASM^ protein was not detected (Fig. 4c). Two independent *Mre11*^cond/ASM^ MEF lines were converted to *Mre11*^−/ASM^ by Cre recombinase introduced via adenovirus (Fig. 4c). When exposed to IR, these cells demonstrated defective activation of ATM (Fig. 4c).

### Low MRE11^ASM^ levels revealed by cellular fractionation

Cells in which MRE11^ASM^ was forcibly expressed showed mislocalization of the protein to cytoplasm. Although no MRE11^ASM^ expressed from the endogenous locus was detected in whole cell extracts, we determined if cellular fractionation might lead to more sensitive detection. We performed the same fractionation as described above and could detect low levels of a protein migrating at the size expected for MRE11^ASM^ (Fig. 4d). This band was present in two independent *Mre11*^+/ASM^ MEF lines, and not in the *Mre11*^+/+^ control. Furthermore, the band was only detected in the cytoplasmic fraction (indicated by * in Fig. 4d lanes 3 and 5), whereas MRE11^+^ protein was present in all fractions. In addition, we also exposed parallel cultures to MG-132, which had caused MRE11^ASM^ expressed from cDNA to relocate to chromatin (Fig. 2b). In the case of MRE11^ASM^ expressed from the endogenous locus, we observed that the band was rendered undetectable in the cytoplasmic fraction (Fig. 4d, lanes 4 and 6). However, unlike the cDNA-driven protein, we did not observe the band appearing in the chromatin fraction (Fig. 4d, lanes 16 and 18). It is unclear whether this represents a true difference in the fate of the proteins encoded in a different manner, or if the presence of a small amount of MRE11^ASM^ protein generates a band too faint to visualize. Nonetheless, the disappearance of the smaller band from the cytoplasmic fraction suggests that inhibition of the proteasome has a similar impact on MRE11^ASM^ encoded from the endogenous locus.

## Discussion

We have found that the transcript resulting from the *MRE11*^ASM^ ATLD17/18 disease allele can be detected as a naturally occurring splice variant in mice, but could not be detected in human cell lines. The impact of this exon skipping maintains the open reading frame but has a severe impact on the protein. Most MRE11^ASM^ is mislocalized to the cytoplasm, undoubtedly contributing to the inability of this protein to support early murine embryogenesis. The splice variant likely does not code for a protein with specific function. In the context of the ATLD17/18 siblings, our studies support the notion that the contribution of MRE11^ASM^ to the disease state is phenotypic resemblance to a null allele. Therefore, the other inherited *MRE11* mutant allele (W243R) likely supports viability of these individuals.

The cellular mislocalization of MRE11 and other components of the MRN complex may be a significant contributor to disease sequelae. In this study we directly compared two different alleles found in ATLD patients; *MRE11*^ASM^ which deletes 27 amino acids toward the middle of the protein, and *MRE11*^ATLD1^ which deletes the C-terminal 78 amino acids^11,15^. While both of these disease-associated forms of MRE11 display some level of cytoplasmic localization, it must be noted that a proportion of wildtype MRE11 is also localized to the cytoplasm. MRE11^ASM^ clearly demonstrated near total mislocalization to the cytoplasm, whereas the proportion of MRE11^ATLD1^ in the cytoplasm resembled that of MRE11^+^. Interestingly, while proteasome inhibition restored chromatin localization of MRE11^ASM^, it did not shift the subcellular localization of MRE11^+^ or MRE11^ATLD1^. This suggests that the proportion of MRE11 normally found in the cytoplasm is distinct from proteasome-mediated mislocalization of pathologic versions of MRE11 such as MRE11^ASM^.

The findings presented here raise the question as to whether the distinct features of MRE11^ASM^ and MRE11^ATLD1^ proteins contributes to the varied outcomes in the two groups of patients. ATLD1 patients have at best a mild cancer predisposition, with no evidence for early childhood predisposition^26^. Mouse models have reinforced this notion, with *Mre11*^ATLD1^ having a strong impact on cancer only in the context of an engineered oncogene driver^27^. In stark contrast, the two juvenile ATLD17/18 siblings died of pulmonary adenocarcinoma, an extraordinarily rare cancer in pediatric populations. It is reasonable to speculate that the differences in the affected proteins have at least some role in different clinic outcomes. However, a full understanding of the relationship between mutation and specific disease aspects will require extensive long-term efforts using complex mouse models and clinical studies following a larger number of patients and their family members.

The common mutation in *NBS1* (657del5) causing Nijmegen breakage syndrome impairs NBS1 interaction with MRE11 and RAD50. In these patients, unlike those with ATLD, MRE11 and RAD50 are present at normal levels, but are partially sequestered in the cytoplasm^6^. Additional structure-function studies on engineered NBS1 mutants support the notion that disruption of NBS1 interaction leaves MRE11/RAD50 mislocalized to the cytoplasm to varying degrees^28^. Indeed, we have shown previously using co-immunoprecipitations that MRE11^ASM^ weakens interaction with NBS1^10^. Therefore, cytoplasmic mislocalization appears to be a final common outcome in situations where NBS1 interaction is weakened, regardless of which MRN component harbors the mutation. It is possible that this relates to a reported interaction between MRN and Hsp90α, a molecular chaperone that controls folding and stability of proteins and regulates assembly of multiprotein complexes^29^. However, the interaction was restricted to NBS1, and inhibition of Hsp90α ATPase activity reduced NBS1 levels, but not MRE11 or RAD50^30^. Clearly, the interplay between protein quality control and DNA damage responses is complex and represents an important area of future study.

In this work we demonstrate that inhibition of the proteasome restores chromatin localization of MRE11^ASM^. This observation supports the notion that an active process sequesters aberrant MRN components in the cytoplasm. The re-localized MRE11^ASM^ protein could not restore ATM activation, which is not surprising given the defective interaction with NBS1^10^. Protein quality control has been studied in detail for certain cellular compartments, in particular the endoplasmic reticulum and cellular membrane^31^. In comparison, little is known about quality control of nuclear proteins, especially in mammalian cells^32^. Proteasomes are abundant in the nucleus, and are implicated in removing proteins that are misfolded due to polyglutamine expansion tracks found to cause neurological disorders such as Huntington’s and others^33^. In the case of MRE11^ASM^, it is likely that misfolding due to the absence of 27 amino acids is recognized by factors involved in nuclear protein quality control, leading to ubiquitination and degradation. Why this leads to mislocalization in the cytoplasm is an important question in protein quality control.

The findings presented here are part of an ongoing effort to understand how differing mutations cause variable outcomes in MRN related disorders, and to uncover therapeutic opportunities that may be tailored to specific mutations. While this study has shed further light on protein stability and turnover in ATLD, unrelated mechanisms leading to low MRN undoubtedly exist. For example, an ATLD-associated allele harboring a C to T mutation at position 1714 of *MRE11* which generates a stop codon at position 571 has been shown to be subject to nonsense-mediated decay (NMD)^34^. This pathway targets mRNAs harboring premature termination codons for degradation before they can be translated into truncated proteins with potentially deleterious effects for the cell. This is distinct from *MRE11*^ASM^ where there is no premature stop codon, and we readily detected the transcript encoded by the endogenous locus. Therefore, patients whose mutations undergo NMD could benefit from inhibition of this process^35^. In contrast, our observation that proteasome inhibition can restore MRE11 localization in some circumstances raises a distinct therapeutic opportunity.

Clinical trials have shown that proteasome inhibitors can be well tolerated. This approach has proven to be an effective treatment for multiple myeloma in which cancer cells undergo apoptosis when overwhelmed by intracellular immunoglobulin protein that is normally turned over^36^. Given that complete absence of any MRN component causes early embryonic lethality, all disease-causing alleles are hypomorphic. While MRE11^ASM^ is too severe to restore function when relocalized to the nucleus, the prospect exists that more minor mutations that cause mislocalization could maintain enough function to benefit from therapeutic relocalization. More broadly, this approach could hold promise for the treatment of genetic disorders caused by misfolded nuclear proteins subject to proteasome-dependent quality control. First however, studies such as this must be undertaken to provide detailed information about the impact of specific mutations.

## Materials and Methods

### Generation of germline *Mre11* alleles

The targeting construct used plasmid pLNtk as the backbone with arms generated by PCR with genomic DNA from 129X1/SvJ mouse cells^37^. Positive selection used G418 and negative selection used gancyclovir (University of Michigan Transgenic Animal Model Core). Double-resistant clones were screened by Southern blot for targeted integration.

### Ethical use of vertebrate animals

All procedures performed in this study involving animals were in accordance with strict ethical standards. The University of Michigan is accredited by the Association for Assessment and Accreditation of Laboratory Animal Care, International (AAALAC, Intl) and the animal care and use program conforms to the standards of “The Guide for the Care and Use of Laboratory Animals”, Revised 2011.

### Growth and analysis of MEFs

MEFs were isolated from day e13.5 embryos and grown in standard culture conditions as described^38^. Primary MEFs were immortalized by transfection with pBsSVD2005 (SV40 large T antigen expression vector). To create cDNA clones, cells were transfected (Lipofectamine 2000, Life Technologies) with mutant MRE11-expressing constructs^10^ and clones were isolated and grown under blasticidin (Life Technologies) selection. Adeno-Cre (University of Michigan Vector Core) at an MOI of 500 was used. MEFs were grown 3 days post-infection and split once prior to plating for experiments. Kinase inhibitors were used as follows: ATM, KU55933 (10 μM for 1 hr, Tocris Biosciences); DNA-PK, NU7026 (20 μM for 2 hrs, Tocris Biosciences). Proteasome inhibition was carried out for 8 hrs in 20 µM MG-132 (Sigma). Where IR treatment is indicated, cells were exposed to a ^137^Cs source.

### Western blots

Whole cell and tissue extracts were prepared in RIPA buffer (150 mM NaCl; 1% NP-40; 0.5% C_24_H_39_O_4_Na; 0.1% SDS; 50 mM Tris-HCl, pH 8.0), protein concentration was determined by BCA assay (Pierce), extracts were resolved by SDS-PAGE and transferred using standard procedures. Primary antibodies were: MRE11 (Cell Signaling, #4895); γH2AX and H2AX (EMD Millipore, #05–636 and #07–627); pKAP1^S824^ (Bethyl, #A300–767A); Vinculin (Cell Signaling, #4650); TOPOI (BD Biosciences, #556597); Cyclin D1 (Santa Cruz, #sc-8396); β-catenin (Santa Cruz, #sc-7199); and GAPDH (Cell Signaling, #2118). Secondary antibodies for western blots were IRDye-conjugated goat anti-rabbit or anti-mouse (Li-Cor Biosciences).

### Biochemical fractionation

Fractionation experiments were carried out as previously described^39^. Treated or mock-treated cells were washed with ice-cold PBS and cell fractionation was carried out by consecutive extractions with increasing detergent concentration. Cell pellets were first resuspended for 5 min on ice in fractionation buffer I (50 mM HEPES, pH 7.5; 150 mM NaCl; 1 mM EDTA; 0.2% IGEPAL CA-630) supplemented with protease inhibitors. Following centrifugation at 1000 × g for 5 min, the supernatant was collected (fraction I, cytoplasmic), and the pellets were washed with the same buffer. The nuclear pellets were further extracted for 40 min on ice with fractionation buffer II (50 mM HEPES, pH 7.5; 150 mM NaCl; 1 mM EDTA; 0.5% IGEPAL CA-630). The extracts were clarified by centrifugation at 16,000 × g for 15 min (supernatant is fraction II, nuclear). The pellets were finally lysed in RIPA buffer (150 mM NaCl; 1% NP-40; 0.5% C_24_H_39_O_4_Na; 0.1% SDS; 50 mM Tris-HCl, pH 8.0), sonicated, and cleared by centrifugation at 16,000 × g for 20 min (fraction III, chromatin). Equal aliquots of each fraction, determined by BCA assay (Pierce), were separated and immunoblotted as above.

### Determination of embryonic lethality

Mice are *Mus musculus* of C57B6/129sv mixed background. χ^2^ analysis was used to determine the significance of any observed differences between the actual and expected results of the cross performed. No blinding, randomization, or power calculation for sample size was performed. The University of Michigan’s University Committee on the Use and Care of Animals (UCUCA) approved all mouse procedures carried out in this study in accordance with an approved protocol.

### Analysis of *Mre11* cDNA

For reverse transcriptase-mediated PCR (RT-PCR), total RNA from MEF cells, human fibroblasts, and HeLa cells was prepared using QIAGEN RNeasy kit according to instructions or from adult mouse tissues using Trizol solution (Life Technologies) followed by reverse transcription (SuperscriptIII, Life Technologies). Murine *Mre11* cDNA was generated using a poly-dT primer in combination with primers located in exon 9 (5′-GGA GAA AGA TGA ACA TGC AGA AG-3′) and exon 11 (5′-CAA ACT TCT GGC TAA AAC GAA GA-3′). Human *MRE11* cDNA was amplified using primers located in exon 9 (5′-TTT CAT GGA GGA TAT TGT TCT AGC-3′) and exon 12 (5′-CTT AAA GTT GTT CCT TCT GAA GGC-3′). Thermocycling conditions for the above reactions entails 30 cycles of 94 °C for 45 sec, 60 °C for 45 sec, 72 °C for 60 sec. For sequencing, full-length *Mre11* cDNA products were cloned in the T-easy vector (Promega) and sequenced.

### PCR-based Genotyping

PCR primers used for distinguishing *Mre11*^+^ from *Mre11*^ASM^ alleles are as follows:

1. 5′-GCT TAG CTT TCT CAG GAA CAG TG-3′
2. 5′-GGA CAC GGA AGA TAA ACA CTC AG-3′

The unique band for each allele is:

*Mre11*^+^ −1110 bp and for *Mre11*^ASM^ is 614 bp

Thermocycling conditions for the above reaction entails 30 cycles of 94 °C for 45 sec, 60 °C for 45 sec, 72 °C for 60 sec.

### Southern blotting

Genomic DNA isolated from embryonic stem cells was digested with NcoI and separated by gel electrophoresis. Gel was soaked in denaturing solution (1.5 M NaCl; 0.5 M NaOH) then transferred to Zeta-Probe membrane (BioRad) in 10X SSC (1.5 M NaCl; 150 mM HOC(COONa) (CH_2_COONa)2 • 2H_2_O) overnight. Probe DNA was generated by PCR (Fwd primer, 5′-AAG GAT AGA GAT CAG AAA GCC ATG T-3′; Rev primer, 5′-AGA CAG ACT GTA TTA AGT TTC AAC TGT GAG-3′) using 129 × 1/SvJ mouse DNA as the template and 35 cycles of the following reaction conditions: 95 °C for 15 sec, 56 °C for 30 sec, 68 °C for 60 sec. The PCR product was cloned into pCRII-Blunt (Life Technologies) according to instructions and probe DNA was released by EcoRI digestion. Probe was labeled with ^32^Pα-dCTP via nick translation (Roche) according to instructions.

### Availability of data and materials

All data generated or analyzed during this study are included in this published article (and its Supplementary Information files). All biological materials generated for this study are available upon request.

## Electronic supplementary material

Supplementary Information
