# Supplementary material for: Reversible mislocalization of a disease-associated MRE11 splice variant product
Source: Sci Rep. 2018 Jul 4;8:10121. doi: 10.1038/s41598-018-28370-5 (PMC6031676; doi:10.1038/s41598-018-28370-5)
Supplement: Supplementary file 1 — Supplementary Information [file 41598_2018_28370_MOESM1_ESM.pdf]

Reversible mislocalization of a disease-associated MRE11 splice variant product

Andrea J. Hartlerode[1], Joshua A. Regal[1,2], and David O. Ferguson[1,*]

Department of Pathology[1], Molecular and Cellular Pathology Graduate Program[2], The University of Michigan Medical School, Ann Arbor, MI 48109-2200. USA

[*]Correspondence should be addressed to:

David O. Ferguson
The University of Michigan Medical School
109 Zina Pitcher Place, BSRB 2067
Ann Arbor, MI 48109–2200
tel (734) 764–4591
fax (734) 763–2162
daviferg@umich.edu

Supplementary Figure S1

a

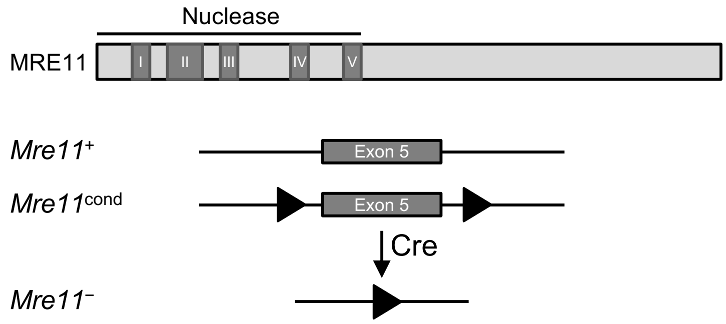

b

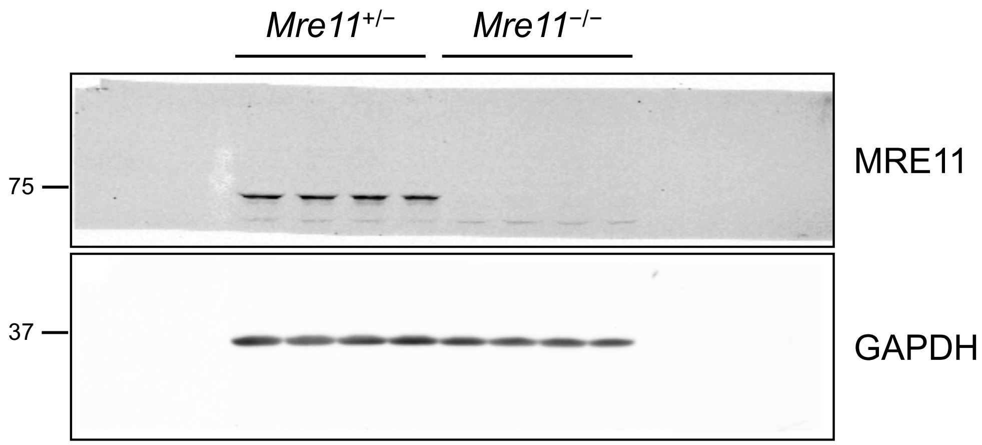

c

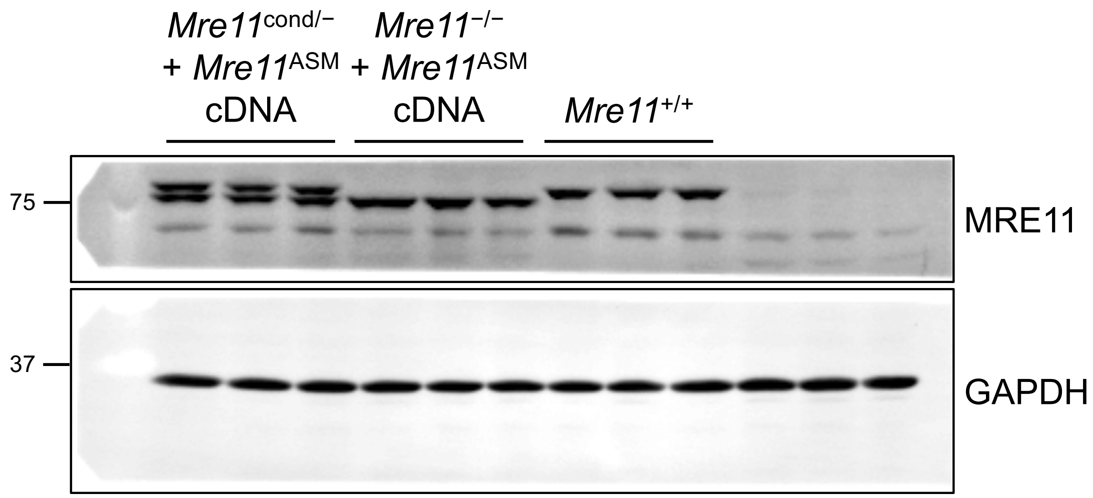

Supplementary Figure S2

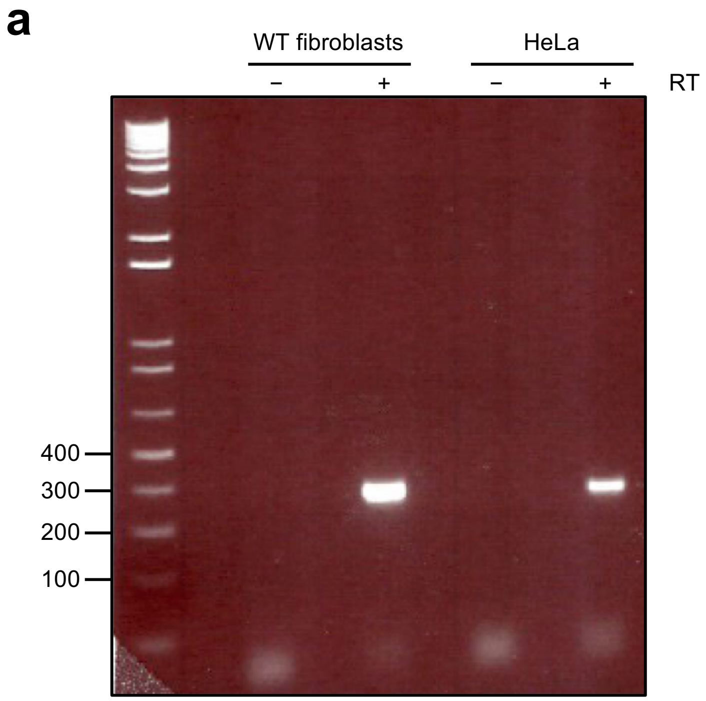

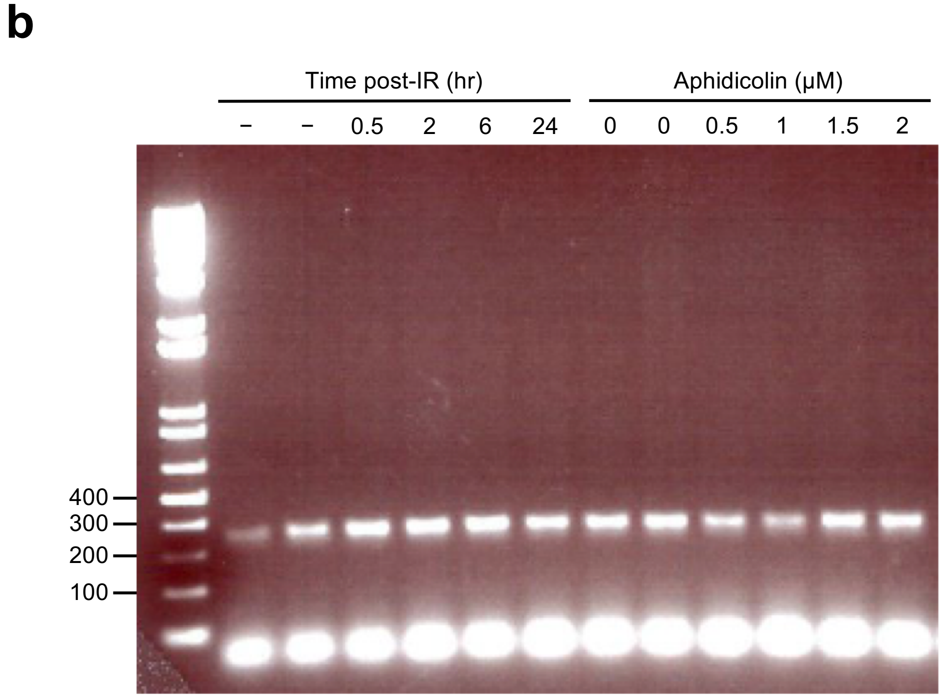

Supplementary Figure S3

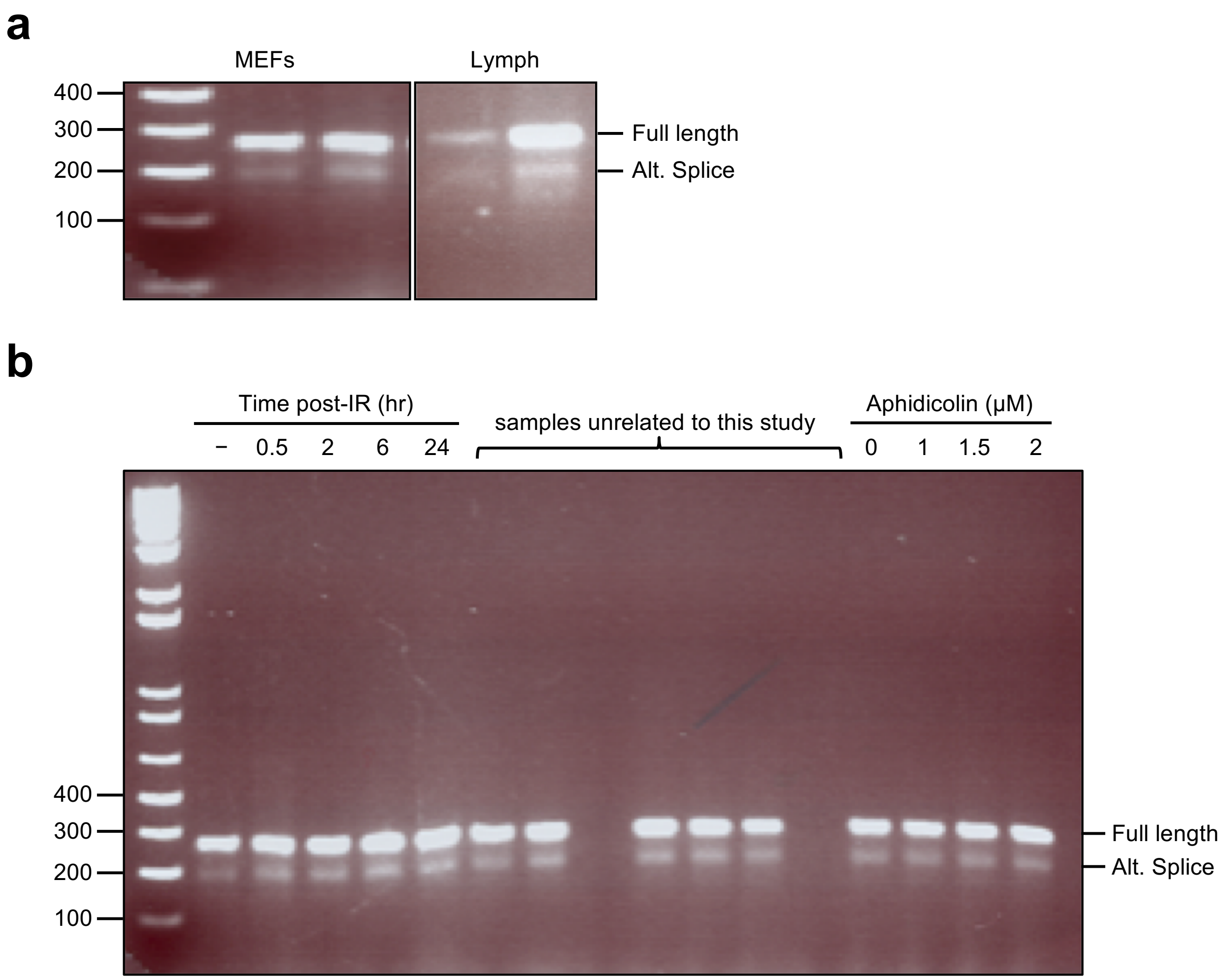

Supplementary Figure S4

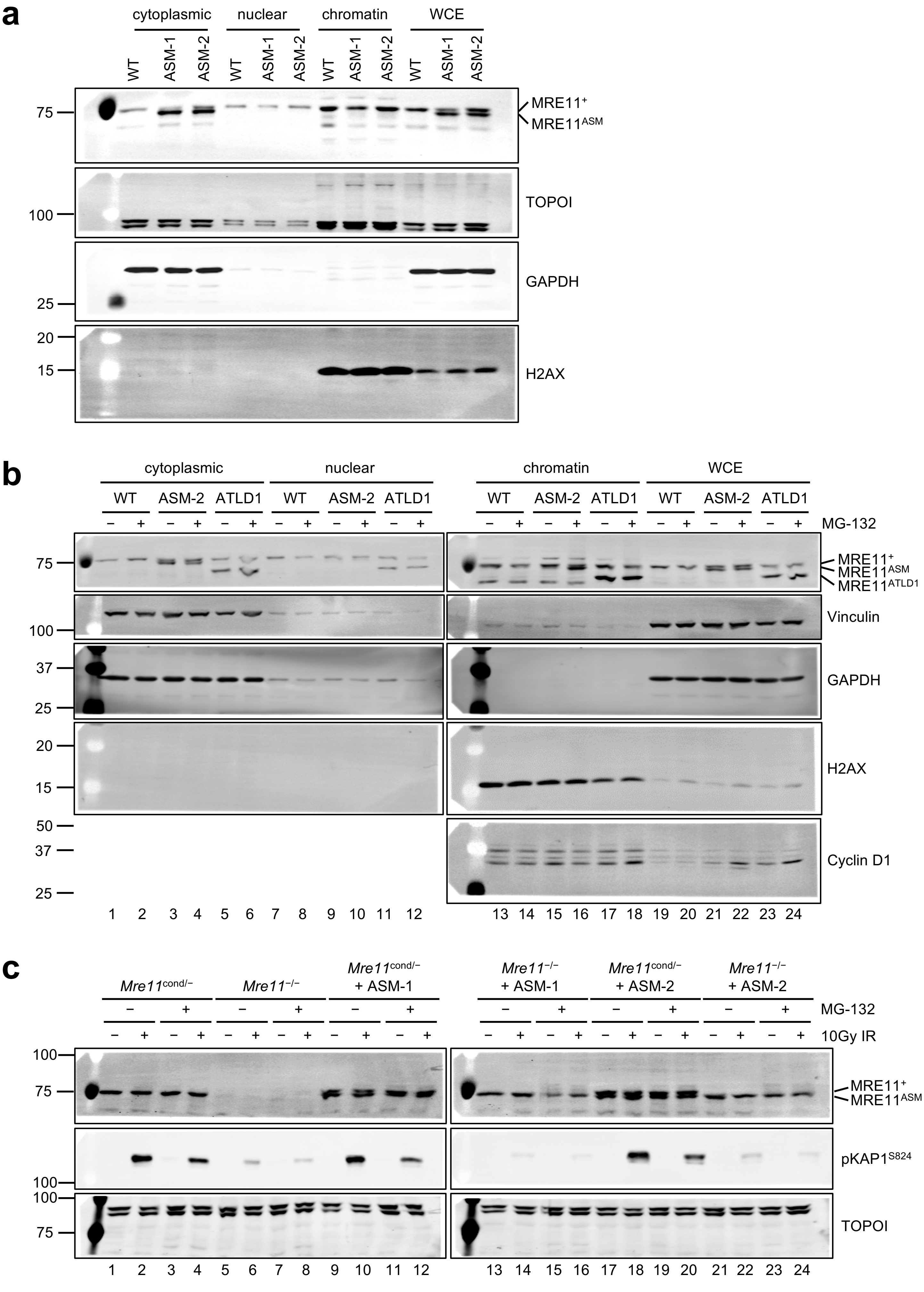

Supplementary Figure S5

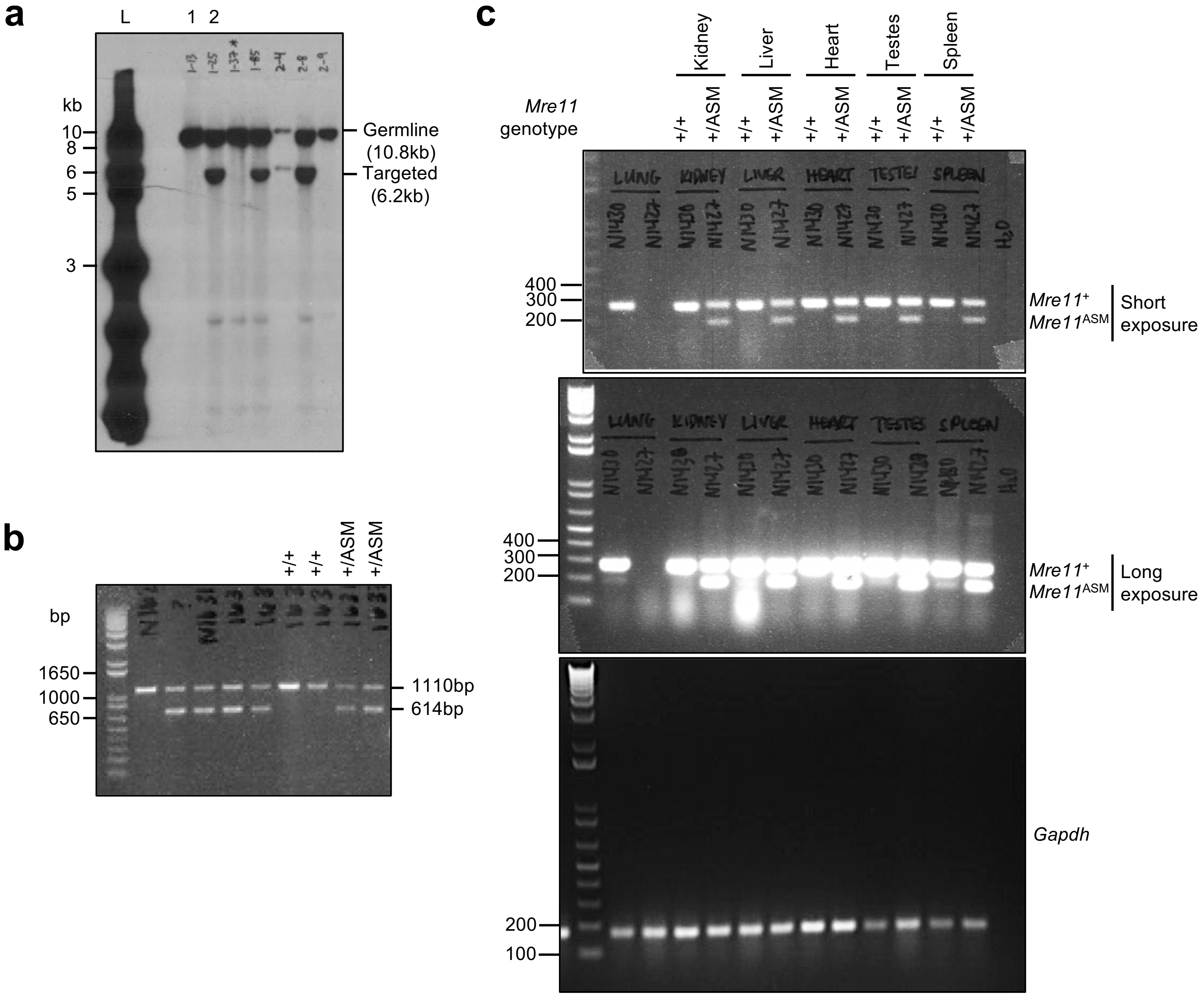

Supplementary Figure S6

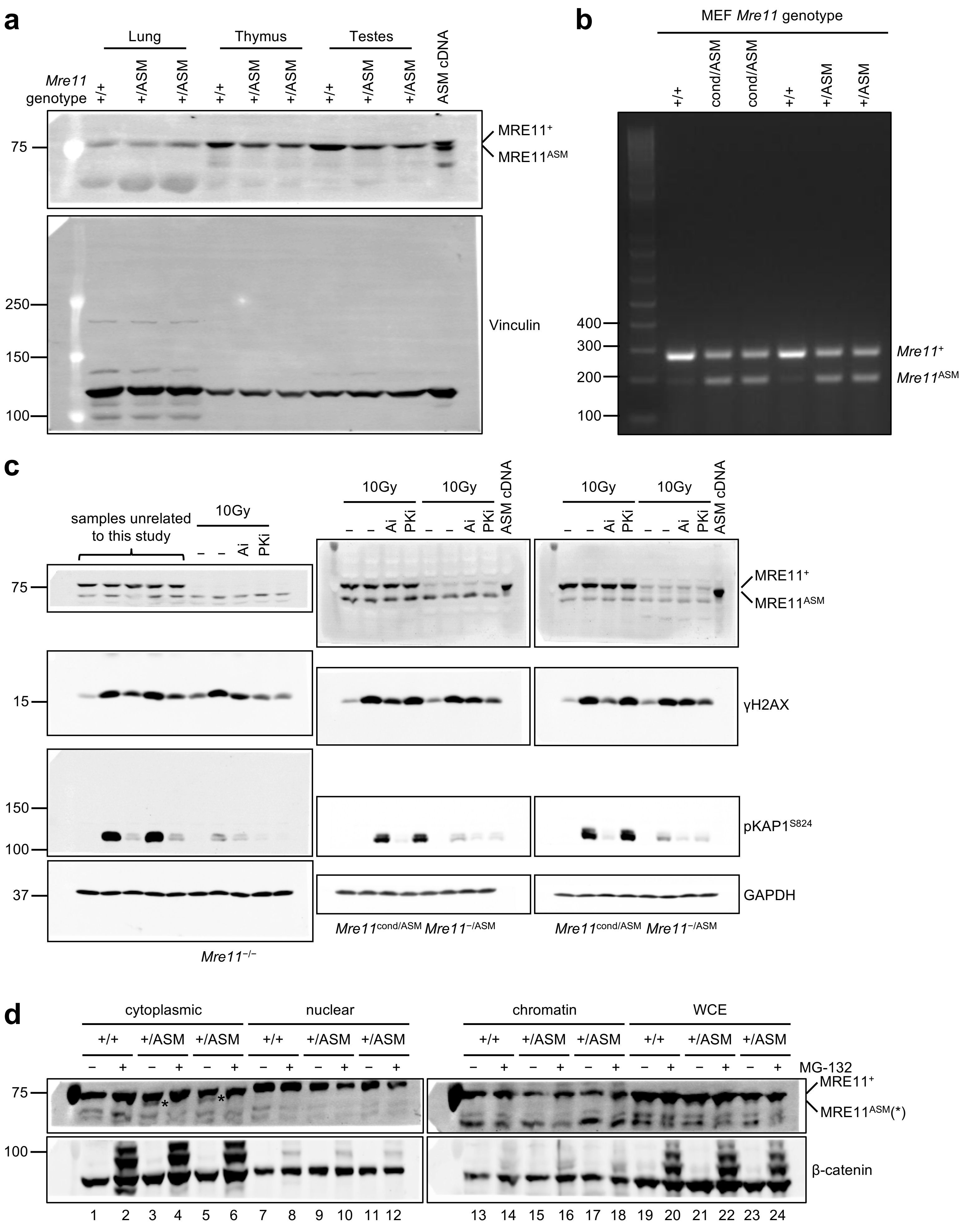

LEGENDS TO SUPPLEMENTARY FIGURES

Supplementary Figure S1 Deletion of endogenous Mre11 using a conditional allele (a) Mammalian MRE11 domain structure (top) and murine germline *Mre11* alleles (bottom). Line, introns; triangles, LoxP sites. (b and c) Immunoblots performed on MEFs, with genotypes at the top, using the indicated antibodies at right. (b) Demonstration of efficient deletion of MRE11 protein using the conditional knockout system. (c) Demonstration of efficient deletion of endogenous MRE11 protein using the conditional knockout system while maintaining physiologic levels of $MRE11^{ASM}$ from an integrated cDNA expression vector.

Supplementary Figure S2 Detection of endogenous $MRE11^{ASM}$ in human cells (a) RT-PCR using a forward primer in exon 9 and a reverse primer in exon 12 performed with or without inclusion of reverse transcriptase (RT) in the reaction (shown at top). A smaller product resulting from exon 10 skipping is not detected in both wild-type human fibroblast or HeLa cell RNA. (b) RT-PCR described in (a) reveals that exposure to ionizing radiation (IR) or aphidicolin does not alter the relative amount of alternatively spliced *MRE11* in HeLa cells.

Supplementary Figure S3 Full-length RT-PCR agarose gel images corresponding to Figure 1.

Supplementary Figure S4 Full-length immunoblot images corresponding to panels in Figure 2.

Supplementary Figure S5 Full-length Southern blot, genotyping, and RT-PCR agarose gel images corresponding to Figure 3.

Supplementary Figure S6 Full-length immunoblot and RT-PCR agarose gel images corresponding to Figure 4.
